# *Microsporidia MB* in the primary malaria vector *Anopheles gambiae *sensu stricto is avirulent and undergoes maternal and horizontal transmission

**DOI:** 10.1186/s13071-023-05933-8

**Published:** 2023-09-25

**Authors:** Godfrey Nattoh, Brenda Onyango, Edward Edmond Makhulu, Diana Omoke, Lilian Mbaisi Ang’ang’o, Luna Kamau, Maxwell Machani Gesuge, Eric Ochomo, Jeremy Keith Herren

**Affiliations:** 1 Centre for Global Health Research (CGHR), Kenya Medical Research Institute, Kisumu, Kenya; 2 Department of Biological Sciences, Kaimosi Friends University, Kaimosi, Kenya; 3 International Centre of Insect Physiology and Ecology (icipe), Nairobi, Kenya; 4 Research Unit in Bioinformatics (RUBi), Department of Biochemistry and Microbiology, Rhodes University, Grahamstown, South Africa; 5 Centre for Biotechnology Research and Development (CBRD), Kenya Medical Research Institute, Nairobi, Kenya

**Keywords:** Symbiosis, *Microsporidia MB*, *Anopheles*, Vectors

## Abstract

**Background:**

The demonstration that the recently discovered *Anopheles* symbiont *Microsporidia MB* blocks malaria transmission in *Anopheles* *arabiensis* and undergoes vertical and horizontal transmission suggests that it is a promising candidate for the development of a symbiont-based malaria transmission-blocking strategy. The infection prevalence and characteristics of *Microsporidia MB* in *Anopheles gambiae* sensu stricto (s.s.), another primary vector species of malaria in Kenya, were investigated.

**Methods:**

Field-collected females were confirmed to be *Microsporidia MB*-positive after oviposition. Egg counts of *Microsporidia MB*-infected and non-infected individuals were used to infer the effects of *Microsporidia MB* on fecundity. The time to pupation, adult sex ratio and survival were used to determine if *Microsporidia MB* infection has similar characteristics in the host mosquitoes *An. gambiae* and *An. arabiensis*. The intensity of *Microsporidia MB* infection in tissues of the midgut and gonads, and in carcasses, was determined by quantitative polymerase chain reaction. To investigate horizontal transmission, virgin males and females that were either *Microsporidia MB*-infected or non-infected were placed in standard cages for 48 h and allowed to mate; transmission was confirmed by quantitative polymerase chain reaction targeting *Microsporidia MB* genes.

**Results:**

*Microsporidia MB* was found to naturally occur at a low prevalence in *An. gambiae* s.s. collected in western Kenya. *Microsporidia MB* shortened the development time from larva to pupa, but other fitness parameters such as fecundity, sex ratio, and adult survival did not differ between *Microsporidia MB*-infected and non-infected hosts. *Microsporidia MB* intensities were high in the male gonadal tissues. Transmission experiments indicated that *Microsporidia MB* undergoes both maternal and horizontal transmission in *An. gambiae* s.s.

**Conclusions:**

The findings that *Microsporidia MB* naturally infects, undergoes maternal and horizontal transmission, and is avirulent in *An. gambiae *s.s. indicate that many of the characteristics of its infection in *An. arabiensis* hold true for the former. The results of the present study indicate that *Microsporidia MB* could be developed as a tool for the transmission-blocking of malaria across different *Anopheles* species.

**Graphical Abstract:**

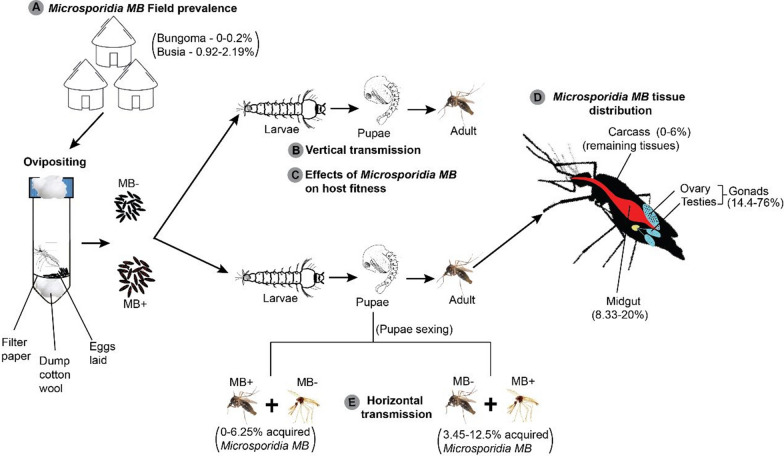

**Supplementary Information:**

The online version contains supplementary material available at 10.1186/s13071-023-05933-8.

## Background

*Anopheles gambiae* sensu stricto (s.s.) mosquitoes are efficient vectors of the main malaria parasite *Plasmodium falciparum*, especially in parts of sub-Saharan Africa, where malaria still remains a huge burden. While frontline insecticide-based vector control strategies have contributed significantly to the decrease in malaria cases [1], there are challenges that need to be overcome, such as the development of insecticide resistance [2, 3]. Alternative mosquito-borne control interventions that utilize symbionts which can be stably maintained in their mosquito hosts, conferring protection against parasites and pathogens, e.g. in *Anopheles* against *P. falciparum* [4–6] and in *Aedes* against arboviruses [7–9], have been proposed. The discovery that the novel *Microsporidia MB* symbiont identified in wild *Anopheles arabiensis* mosquitoes protects these hosts against infection with *Plasmodium* parasites [10] and is vertically and horizontally transmitted in adult mosquitoes [11], has led to the suggestion that it could be a promising candidate for integrated vector management.

While *Microsporidia MB* was initially reported in *Anopheles arabiensis*, which is a member of the *Anopheles gambiae* sensu lato (s.l.) species complex, there have been reports from Ghana that it infects other members of the *An. gambiae* species complex, such as *Anopheles coluzzii* and *An. gambiae* s.s. [12]. These findings are highly significant since *An. gambiae* s.l. is the main malaria vector in many parts of sub-Saharan Africa [13–15]. As *An. arabiensis* and *An. gambiae* s.s. are closely related and often found in sympatry [16], it is possible that *Microsporidia MB* could have similar characteristics in both of them, including vertical transmission, a lack of sex bias, and avirulence. In the wild, *Microsporidia MB* is generally found at a prevalence that ranges from 0 to 9% in populations of *Anopheles arabiensis, Anopheles funestus, Anopheles gambiae,* and *Anopheles coluzzii* [10–12]. Although *Microsporidia MB* has been reported in diverse *Anopheles* species, the infection characteristics in species of this genus other than those mentioned above have not yet been investigated. *Anopheles* mosquitoes collected from western Kenya, where *An. gambiae* s.s. is the dominant species, were used to determine the prevalence of *Microsporidia MB* infection in this host. Further, it was investigated if the infection characteristics of *Microsporidia MB* in *An. gambiae* s.s. are similar to those previously observed in *An. arabiensis.*

## Methods

### Ethical clearance

The Kenya Medical Research Institute (KEMRI) undertook an ethical review and granted ethical approval for this work [KEMRI/Scientific and Ethics Review Unit (SERU)/Centre for Biotechnology Research and Development (CBRD)/230/4341]. Community leaders and village participants enrolled for sample collection were provided with a detailed explanation of the study objectives, activities, procedures, benefits and risks before the commencement of data collection. Through the community leaders, informed consent was obtained from the owners of the households where samples were collected. Participation was on a voluntary basis and participants were free to withdraw from the study at any point.

### Description of archived *Anopheles* samples

*Anopheles* mosquito DNA samples used for screening *Microsporidia MB* were retrieved from various projects undertaken at the Centre for Global Health Research in KEMRI between 2019 and 2021. Protein precipitation was used to extract nucleic acids (Puregene; Qiagen, the Netherlands). The DNA samples were from individual adult mosquitoes previously collected from different sites across Busia and Bungoma in western Kenya. The species were confirmed by molecular assay. To assess DNA integrity, a subset was selected at random to determine the quantity and quality of DNA by using a NanoDrop 2000c Spectrophotometer (Thermo Fisher, UK). An average DNA concentration of 61.3 ng/µL (25.73–184.23 ng/µL) and purity of 1.7 (1.42–2.10) were obtained.

### Field collection of *Anopheles* mosquitoes

Collections of resting gravid and engorged female mosquitoes were undertaken indoors using manual aspiration. Collection in Busia (− 34.105278W, − 0.463333N) was undertaken between August 2021 and February 2022 from 0700 to 1130 hours using aspirators and electric torches/lights to target endophagic and endophilic gravid individuals that had acquired a blood meal late at night and were likely to be found in crevices or dark corners of the houses [15]. Similarly, collection was by indoor catches, undertaken from 0600 to 0900 hours, in Busia and areas of Bungoma adjacent to where previous studies had found more of the highly anthropophilic *An*. *gambiae* s.s. and *An*. *funestus* s.l. than *An*. *arabiensis* [17, 18]. Sites in Bungoma were excluded from the planned field collection where archived samples showed a low prevalence of *Microsporidia MB* compared to those from Busia. Collected females were placed in netted paper cups or standard cages and supplied with 10% sucrose before transporting them to KEMRI/Centre for Global Health Research insectary laboratory for processing.

### Generating *Microsporidia MB-*infected semi-field colonies

All wild-caught mosquito females were identified morphologically using the keys of Gillies and Coetzee [19]. In the areas of Busia where mosquitoes were collected, *An*. *gambiae* s.l. and *An*. *funestus* s.l. are the commonest *Anopheles* species, with > 93% of the members of the *An*. *gambiae* species complex there previously confirmed as *An. gambiae* s.s. by PCR [18].

The females were placed in individual tubes containing a wet 1-cm × 1-cm Whatman filter paper to induce oviposition, and maintained in an insectary at 28 ± 2.5 °C, relative humidity of 60–80%, under a 12-h day and 12-h night cycle. All of the eggs laid by the *Microsporidia MB-*infected and non-infected females were counted under a microscope and placed in trays containing water for larval development in a semi-field setup at 30 ± 2.5 °C and relative humidity of 30–40% [11]. Once they had laid eggs, the generation zero (G_0_) females were set aside for processing to extract DNA, re-confirm their species identity, and screen them for the presence of *Microsporidia MB* infection, as previously described [10, 11]. The larvae of the *Microsporidia MB*-infected females were reared in pools (76% of samples, where the offspring of seven females constituted one pool), or as individual broods (24% of samples). The larvae were first fed 0.1 mg TetraMin Baby/larva per day, which was increased to 0.3 mg/larva per day for the late-stage instars before pupation. Adults were maintained in separate cages according to brood or pool and supplied with 10% sucrose. Field-derived G_1_ non-infected broods or pools were reared under the same conditions but separate from the *Microsporidia MB*-infected individuals, and were used as control isofemale lineages.

### Determination of the effects of *Microsporidia MB* on *An. gambiae* s.s. fitness

To determine the effects of *Microsporidia MB* on host life history traits, 2464 wild-caught gravid female mosquitoes were placed in perforated 1.5-mL microcentrifuge tubes containing a soaked piece of Whatman paper to induce oviposition. The eggs from each female were counted as previously described [10, 11] before placing them in tubes containing water. After egg-laying, 53 G_0_ females that were confirmed to be *An. gambiae* s.s. and *Microsporidia MB*-positive by using qPCR were used to produce *Microsporidia MB*-positive progeny alongside non-infected controls selected at random (*n* = 374). The offspring were used to determine (i) the time period of larval development until pupation; (ii) the sex ratio, by counting the number of males and females in the broods of individual or pooled females; (iii) adult survival; and (iv) vertical transmission efficiency, by determining the number of offspring acquiring *Microsporidia MB* from each wild-caught *Microsporidia MB*-infected female.

### Transmission of *Microsporidia MB* between live *An. gambiae* s.s. adults

*Microsporidia MB*-infected and uninfected *An. gambiae* s.s. virgin adult offspring were reared in standard 30-cm × 30-cm × 30-cm cages. Virgin male and female pupae were determined by visual examination of the terminalia and hatched in separate cages, as previously described [11]. To increase the chance of transmission, individual *Microsporidia MB*-infected donor and non-infected male and female recipients of the same age (range, 5–7 days old) were kept together in cages for 48 hr. The number of *Microsporidia MB*-infected donors of the same age ranged from two to 21 individuals, while those of the virgin uninfected recipients of the opposite sex ranged in number from 12 to 44 individuals (Additional file 4: Table S2). The set up for mates of the same age was in duplicate, and each age group represented an independent experiment. As the horizontal transmission of *Microsporidia MB* previously observed in *An. arabiensis* was between individuals of the opposite sex [11], in the present experiment, horizontal transmission was only examined between females and males and vice versa. All of the *An. gambiae* s.s. mosquitoes were subsequently screened by qPCR to establish *Microsporidia MB* infection statuses and intensities in both donor and recipient *An. gambiae* s.s. after the spermathecae had been examined to check for the presence of sperm in the recipient females [11]. A representative number of individuals in a cage (*n* = 5) was checked prior to screening all of the recipient and donor individuals in the cages for *Microsporidia MB* infection by qPCR. All of the samples in an experimental cage were used to determine the number of donors infected with *Microsporidia MB* and the number of recipient individuals acquiring a *Microsporidia MB* infection.

### *Microsporidia MB* in tissues of *An. gambiae* s.s.

*Microsporidia MB* were quantified in dissected tissues of G_1_* Microsporidia MB*-infected *An. gambiae* s.s. adults, 5–7 days post-emergence. Tissues of the midgut and gonads were separated from the whole mosquito, and the remainder was designated “the carcass,” as previously described [11]. The individual tissues were labeled separately and screened for the presence and intensity of *Microsporidia MB* by qPCR. Genomic DNA was extracted from the tissues in individual tubes by using protein precipitation [10, 11].

### Molecular screening and quantification of *Microsporidia MB*

The detection and quantification of *Microsporidia MB* were done with specific primers (MB18SF, CGCCGGCCGTGAAAAATTTA; MB18SR, CCTTGGACGTGGGAGCTATC) previously designed to detect *Microsporidia MB* in *An*. *arabiensis* [10, 11]. Briefly, a 10-µL polymerase chain reaction (PCR) master mix consisting of 2 µL HOT FIREPol Blend Master Mix Ready to Load (Solis BioDyne, Estonia; mix components included HOT FIREPol DNA polymerase, 2 mM of each deoxynucleoside triphosphate and 7.5 mM magnesium chloride), 5 µL of nuclease-free PCR water, 0.5 µL of 5 pmol µL^−1^ forward and reverse primers, and 1 µL of the sample template, was prepared. The mixture was incubated in a thermocycler set up as follows: initial denaturation at 95 ˚C/15 min, followed by 35 cycles of denaturation at 95˚C/60 s, primer annealing for 90 s at 62 ˚C, extension at 72 ˚C for 60 s, and a final chain elongation step of 72 ˚C for 5 min. A qPCR reaction carried out using the MB18SF/MB18SR primers on a MIC qPCR cycler (Bio Molecular Systems, Australia) was used to determine the intensity of *Microsporidia MB* infection. These data were normalized by using *Anopheles* host ribosomal S7 gene primers (S7F, TCCTGGAGCTGGAGATGAAC; S7R, GACGGGTCTGTACCTTCTGG) [21].

### Statistical analysis

An unpaired* t*-test was used to analyze data that were assumed to be normally distributed, while non-normally distributed data were analyzed by either two-tailed unpaired Mann–Whitney *U*-test or Kruskal–Wallis test following Dunn’s multiple comparisons post hoc test. A Mann–Whitney *U*-test was used to compare fecundity and development time between *Microsporidia MB*-infected and non-infected mosquitoes, while the Kruskal–Wallis test was used to estimate the significance of *Microsporidia MB* infection in tissues. To measure the level of significance of *Microsporidia MB* positivity rates, a chi-square test was used, while a log-rank test was used to estimate survival. A linear regression was used to establish the correlation coefficient between G_o_ and G_1_ infection intensities and prevalences. To examine whether *Microsporidia MB* intensities impacted transmission rate, a mixed-effect model fit by maximum likelihood was used, where the total individuals exposed and the number of donors in the cage were considered random intercepts. All of the analyses were performed using either R (version 4.1.2) or GraphpadPrism software. The data are presented as mean ± SEM, and *P* < 0.05 was deemed to indicate statistical significance.

## Results

### *Microsporidia MB* detected in *An. gambiae* s.s.

**Fig. 1:**
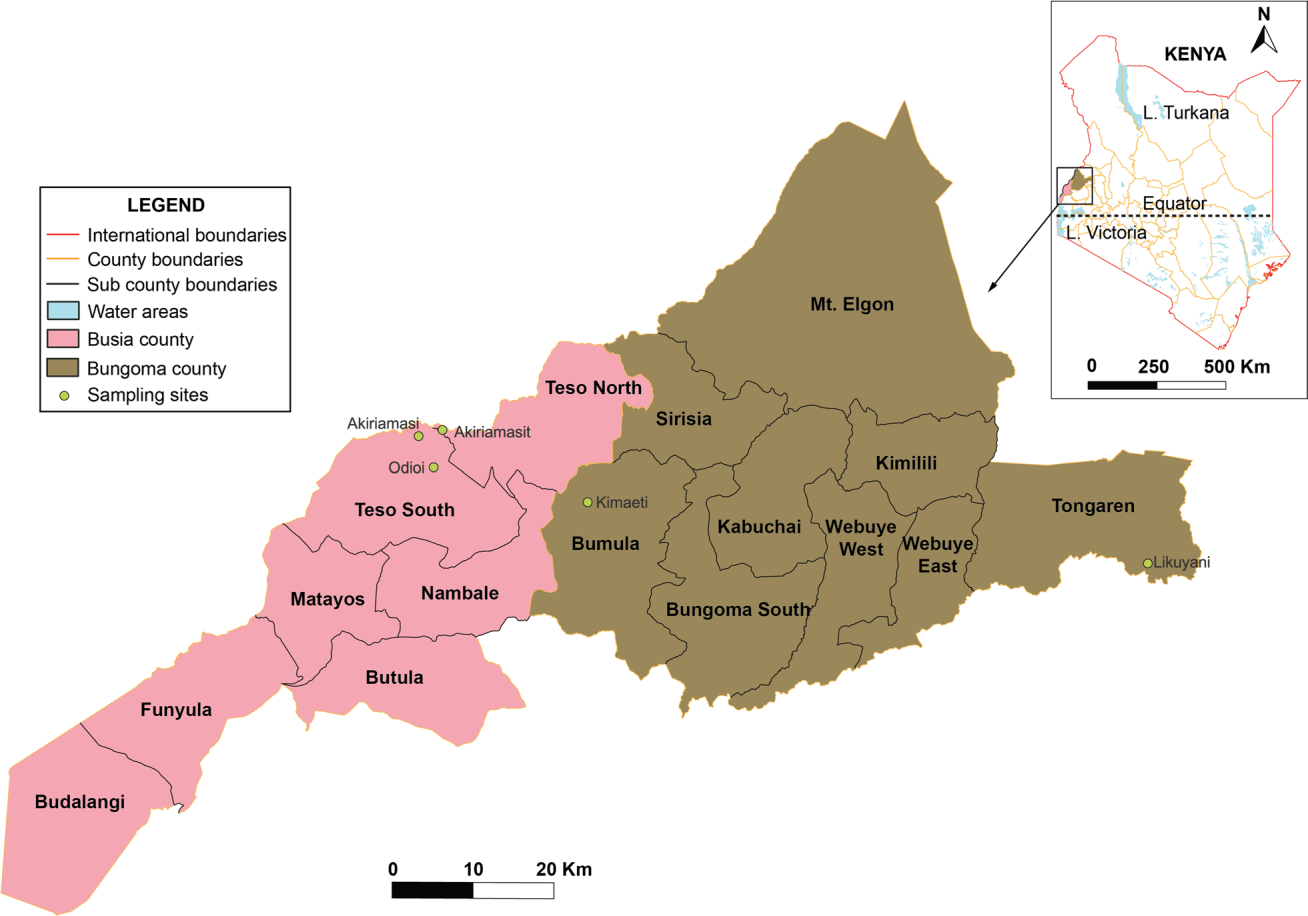
Map showing the sampling sites in Busia and Bungoma, western Kenya, where *Anopheles gambiae* sensu lato (s.l.) infected with *Microsporidia MB* were collected

**Fig. 2:**
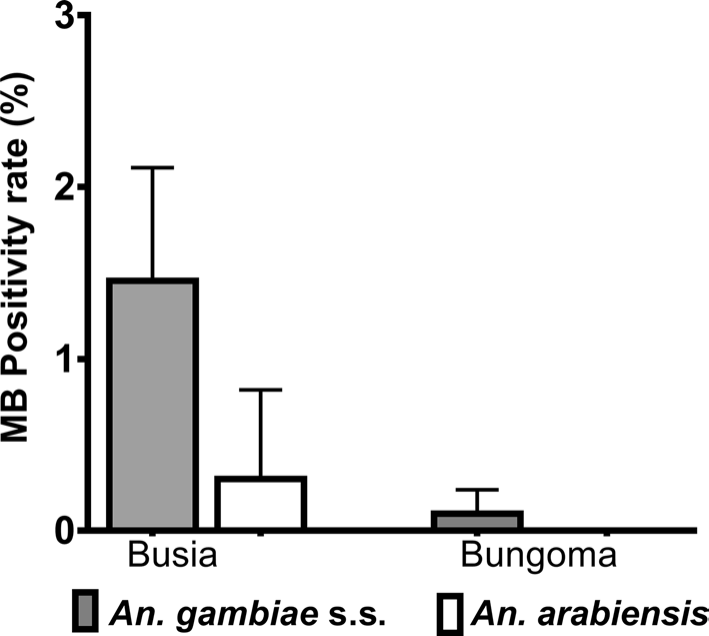
Positivity rate of *Microsporidia MB* (*MB*) infection in *Anopheles gambiae* sensu stricto (s.s.) and *Anopheles* *arabiensis* collected from the Busia and Bungoma study sites. Bar plots represent the prevalence of *Microsporidia MB* in *An. gambiae* s.s. and *An. arabiensis* from Bungoma and Busia. Error bars represent the SEM (* *P* < 0.05)

*Microsporidia MB* was found in 79 out of 5067 DNA samples from mosquitoes collected from the two areas in western Kenya (Fig. 1). The overall *Microsporidia MB* prevalence was 1.7% (0.92–2.19) and 0.2% (0–0.2) for the total number of samples analyzed from Busia (78/4561) and Bungoma (1/506), respectively (Fig. 2; Additional file 3: Table S1). The average prevalence of *Microsporidia MB* in *An. gambiae* s.s. from the two areas was 1.6% (79/5067), and ranged from 0 to 2.2%. These prevalences are lower than those previously observed in sibling species of the *An.* *gambiae* species complex, such as *An. arabiensis* [10], *An. gambiae* s.s. and *An. coluzzii* [12]. *Microsporidia MB* was not detected in *An. funestus* s.s. from Busia (0/769) or from Bungoma (0/665) (Additional file 3: Table S1). *Microsporidia MB* was detected in *An. arabiensis* from Busia at a prevalence of 0.9% (3/333), but none of the* An*. *arabiensis* individuals from Bungoma harbored *Microsporidia MB*. *Anopheles gambiae* s.s*.* from Busia accounted for 98.7% (78/79) of individuals of this species harboring *Microsporidia MB*, while *An. gambiae* s.s. from Bungoma accounted for 1.3% of these mosquitoes (1/79), with only one individual harboring *Microsporidia MB*. The 78 *Microsporidia MB-*infected *An. gambiae* s.s*.* from Busia were from 4561 (1.7%) of the mosquitoes collected there; the single infected *An. gambiae* s.s*.* from Bungoma was from 506 (0.2%) mosquitoes collected there.

For Busia, 80.5% (4561/5663) of the collected *Anopheles* mosquitoes were identified as *An. gambiae* s.s., while 13.6% (769/5663) were identified as *An. funestus* s.s., and 5.9% (333/5663) were confirmed as *An. arabiensis*. In Bungoma, *An. gambiae* s.s. accounted for 43.2% (506/1171) of *Anopheles* mosquitoes collected there, while *An. funestus* s.s. was the predominant species at 56.8% (665/1171): *An. arabiensis* was not detected in this area.

When samples collected in 2022 were included in the analysis, the rate of *Microsporidia MB* positivity differed significantly between the two areas [χ^*2*^(1, *n* = 5067) = 6.8, *P* = 0.00917]. There was a lack of statistical significance when the data for the mosquitoes collected in 2020 were analyzed separately [χ^*2*^(1, *n* = 1818) = 2.6968, *P* = 0.10055] or were combined with those from the 2021 collection where *Microsporidia MB* was not detected [χ^*2*^(1, *n* = 2603) = 3.8529, *P* = 0.4966]. The results from the analysis of archived samples collected in 2020 and 2021 informed the subsequent field collection of *An. gambiae* s.s*.* from Busia (*Microsporidia MB* prevalence of 1.1%), where the *Microsporidia MB* positivity rate was higher than that in Bungoma (*Microsporidia MB* prevalence of 0.2%). For the 2022 field collections, *Microsporidia MB* was detected in *An. gambiae* s.s*.* (54/2464), and *An. arabiensis* (3/281) from Busia, but not in *An*. *funestus* s.l. (0/181).

Fifty-three of the 54 *Microsporidia MB*-positive *An. gambiae* s.s. that laid eggs were used to generate F1 progeny together with 374 non-infected *An. gambiae* s.s., to explore the life history traits of *Microsporidia MB*-infected *An. gambiae* s.s. and the transmission characteristics of *Microsporidia MB* in them.

### *Microsporidia MB* is avirulent in *An. gambiae* s.s.

**Fig. 3:**
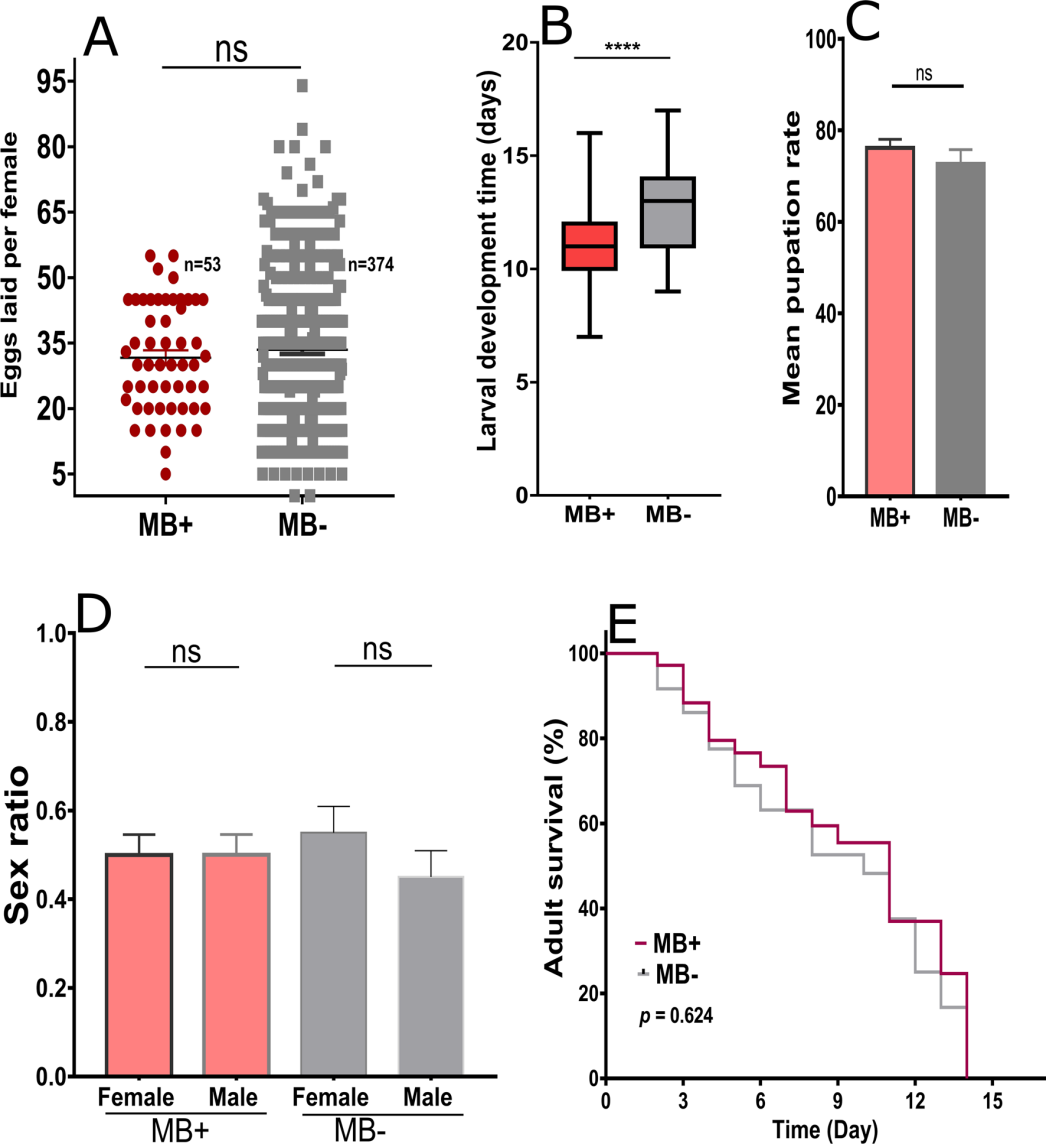
*Microsporidia MB* did not affect certain metrics of *Anopheles gambiae *s.s. fitness. **a** Individual values; the line represents the mean number of eggs laid by females infected with *Microsporidia MB* (*MB+*) (*n* = 53) and by uninfected females (*M−*) (*n* = 374). Bar plots representing mean larval development time (**b**) and mean pupation rate (**c**) of *Microsporidia MB*-infected generation 1 (G_1_) and non-*Microsporidia MB*-infected G_1_. **d** Bar plots representing the mean sex ratio of the progenies of *Microsporidia MB*-infected and non-infected counterparts. **e** Survival curves representing the longevity of adult *An. gambiae* s.s. infected with *Microsporidia MB* and non-infected individuals. The results are expressed as mean ± SEM of independent experiments done in triplicate. *ns* No significant difference, **** P < 0.0001

To determine the characteristics of *Microsporidia MB* infection in *An*. *gambiae* s.s., parameters such as fecundity, larval development time, sex ratio, and adult survival were investigated in infected vs. uninfected isofemale lineages. The number of eggs laid by *Microsporidia MB*-infected females (*n* = 53) versus uninfected individuals (*n* = 374) did not differ significantly (two-tailed Mann–Whitney *U*-test = 9778, *P* = 0.874; Fig. 3a), indicating that *Microsporidia MB* does not have a sterilizing effect on female *An. gambiae* s.s. A significantly shortened larval development time was observed in *An. gambiae* s.s. larvae infected with *Microsporidia MB* (unpaired two-tailed *t*-test, *t* = 2.023, *df* = 106, *P* < 0.0001; Fig. 3b). No statistically significant difference was observed in the mean pupation rate between *Microsporidia MB-*infected (76.14 ± 1.88) and uninfected (72.68 ± 3.10) *An. gambiae* s.s. (unpaired two-tailed *t*-test, *t* = 0.955, *df* = 17, *P* = 2.11; Fig. 3c). The proportion of male and female offspring from *Microsporidia MB*-infected and uninfected *An. gambiae* s.s. did not differ significantly in isofemale lineages (unpaired two-tailed *t*-test, *t* = 0.1712, *df* = 22, *P* = 0.8657; Fig. 3d) or pooled lineages (Mann–Whitney *U*-test = 17, *P* = 0.9026; Additional file 1: Fig. S1A). No statistically significant differences were observed in survival between adult *An. gambiae* s.s. infected with *Microsporidia MB* and those that were uninfected (two-sided log-rank Mantel–Cox, *χ*^2^ = 0.2406, *df* = 1, *P* = 0.624; Fig. 3e). Taken together, these findings suggest that *Microsporidia MB* harbored in *An. gambiae* s.s. is avirulent.

### *Microsporidia MB* undergoes vertical and horizontal transmission in *An. gambiae* s.s.

**Fig. 4:**
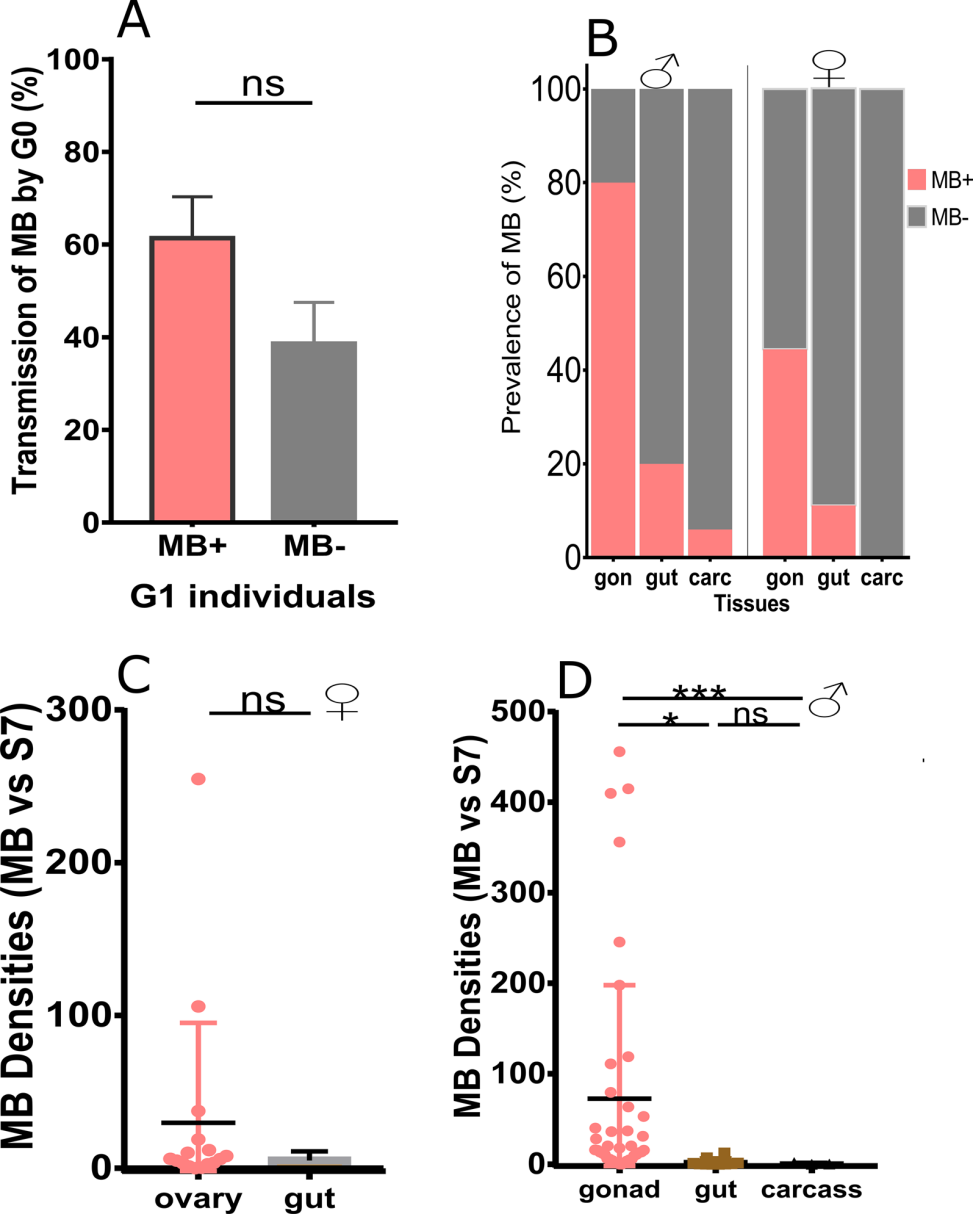
Transmission of *Microsporidia MB* from *Anopheles gambiae* s.s. to their offspring. **a** Bar plots represent transmission rate of *Microsporidia MB* from 12 infected field-derived females (G_o_; *n* = 12) to their offspring (G_1_). **b** Bar plots representing *Microsporidia MB* prevalence in tissues of male and female *An. gambiae* s.s. [gonads (*gon*), guts (*gut*) and carcass (*carc*)]. Scatter plots representing mean relative densities of *Microsporidia MB* transmitted to *An. gambiae* s.s. tissues (gonads, guts and carcass) of female offspring (**c**) and gonads and guts of male offspring, but not male carcasses (**d**). The results are expressed as the mean ± SE from three separate experiments. * P < 0.05, ***P < 0.001

*Microsporidia MB* transmission from field-derived females to their offspring was observed at a frequency ranging from 0 to 100% (61.4 ± 8.93%) in 12 isofemale lineages (Fig. 4a**)** and from 28.6 to 85.3% (59.91 ± 8.46%) in six pooled lineages (Additional file 1: Figure S1B), which led to the hypothesis that *Microsporidia MB* colonizes and proliferates in the reproductive tissues of *An. gambiae* s.s.

 To gain insights into the distribution of *Microsporidia MB* infecting *An. gambiae* s.s. host tissues, such as the midgut and reproductive tissues, and remaining sections (referred to as the carcass here), 7- to 10-day-old G_1_ adults were dissected and screened for the presence and intensity of *Microsporidia MB*. The prevalence of *Microsporidia MB* was high in the gonads of both females (84.21%, *n* = 36) and males (74.51%, *n* = 50) compared to female guts (15.79%) and males guts (19.61%) and males carcasses (5.88%) (Fig. 4b). The intensity of *Microsporidia MB* infection in female reproductive tissue was relatively high, but did not differ significantly from that in the guts (Mann–Whitney *U-*test = 24, *P* = 0.494, *n* = 17; Fig. 4c). The intensity of *Microsporidia MB* infection was significantly higher in the male gonads than in the male guts [χ^2^(2) = 135.265, *P* < 0.05, *n* = 50]; Fig. 4d) and in male gonads compared to male carcasses [χ^*2*^(1, *n* = 36) = 27.3103, *P* < 0.05; Fig. 4d]. A weak non-significant positive correlation was observed between the intensities of infection in the G_1_ and G_o_ [*r*^2^ = 0.1276, *P* = 0.268, number of broods tested = 18; Additional file 2: Figure S2A]. A significant positive correlation was observed between G_o_ infection intensities and the transmission rate of *Microsporidia MB* to the G_1_ offspring (*r*^2^ = 0.625, *P* = 0.0056, *n* = 18; Additional file 2: Fig. S2B), which suggested the likelihood of intensity-dependent maternal transmission.

When virgin *Microsporidia MB*-positive and non-infected individuals of the opposite sex were kept together in cages, 39 out of 40 *Microsporidia MB*-positive *An. gambiae* s.s. males were able to infect at least one female in their cages (range 3.45–12.5%); these results were confirmed by the visual detection of sperm in the female spermathecae before the validation of *Microsporidia MB* infection by qPCR. For instance, in BUR_KSMS 11 and 12, the spermathecae of three *Microsporidia MB*-infected individuals that contained sperm were confirmed by qPCR (Additional file 4: Table S2). The number of individuals acquiring *Microsporidia MB* infection through horizontal transfer determined by a similar molecular methodology ranged from 6.38 to 12.5% (Additional file 4: Table S2). It is also noteworthy that the transfer of *Microsporidia MB* from infected female(s) to one uninfected male was observed (rate of 6.25%; Additional file 4: Table S2). The lack of a statistically significant difference between infection and successful transmission when the mixed-effect model was applied to the data (*P* > 0.05) suggests that the transmission of *Microsporidia MB* from the reproductive tissues of *An. gambiae* s.s. to their offspring is a complex process.

## Discussion

The recent discovery of a *Microsporidia MB* symbiont in *An. arabiensis* with *Plasmodium*-transmission blocking capabilities has generated interest in the potential for the development of a symbiont-based alternative malaria control strategy. Other characteristics of *Microsporidia MB*, such as its transovarial transmission [10], horizontal transmission [11] and avirulence are of high relevance for its potential as a symbiont for the transmission blocking of *Plasmodium*, since these contribute to its ability to spread through and be maintained in populations of *Anopheles* mosquitoes. *Microsporidia MB* has also been found in *Anopheles* species other than *An. arabiensis* [11, 12]; however, the characteristics of these infections and their similarity to those of *Microsporidia MB* in *An. arabiensis* have not been investigated.

The present study demonstrates that *Microsporidia MB* can naturally infect *An. gambiae* s.s. in Kenya, and undergoes transovarial and horizontal transmission without having a deleterious effect on host fitness parameters such as larval development time, pupation rates and adult longevity. These parameters were compared between *Microsporidia MB-*infected and non-infected *Anopheles*, but it could not be ascertained if there were relationships between *Microsporidia MB* infection intensity and these parameters.

The rates of *Microsporidia MB* infection in the populations of *An. gambiae* s.s. in Busia (~ 1.4%) and Bungoma (0.3%) were low. Several factors may explain the observed variations in the number of *An. gambiae* s.s. in the total number of mosquitoes collected from Busia and Bungoma. First, it is likely that seasonality (e.g., the dry and wet seasons in 2020, 2021 and 2022) played a role in the overall abundance of *An. gambiae* s.s. These highlands areas experience bimodal patterns of rainfall, with peak malaria transmission associated with the long wet season from April to July, while the short wet season is from October to November. Low malaria transmission associated with low vector abundance has been reported for the dry season, which lasts from January to March [22]. Second, as the *An. gambiae* species complex exhibits great phenotypic plasticity, with a diversity of resting locations and opportunism in blood feeding [15], the collection methods used for the archived samples, such as indoor aspiration and CDC light traps, may have contributed to variation in the determined species composition. Collection methods that solely target mosquitoes that rest indoors can miss individuals that acquire their blood meal indoors but rest outdoors, which may partly explain some of the variation in the total number of individuals of different species captured in this study.

It is notable that the infection rates observed for *An. arabiensis* were as low as those reported for certain sites in other studies [10]. This suggests that low prevalence could be related to the characteristics of collection sites rather than those of the species of the *An. gambiae* species complex per se. It is, however, notable that the prevalence rates of *Microsporidia MB* agree with those recently reported for *An. gambiae* s.s. and *An. coluzzii* (~ 1.8%) from Ghana [12]. Overall, these findings indicate that *Microsporidia MB* does naturally infect *An. gambiae* s.l. in East Africa. This is significant because *An. gambiae* s.s. remains an extremely important vector of malaria [13, 15, 20]. Of the three main malaria vectors in western Kenya—*An. arabiensis*, *An. gambiae* s.s. and *An. funestus* s.s.—the latter two predominate in the malaria endemic highlands of western Kenya, and are efficient vectors of malaria due to their endophilic and anthropophilic behaviors [15, 22–25].

The collection of fewer *An. arabiensis* in the two areas where sampling was carried out (Additional file 3: Table S1) could be attributed to bias due to our sample collection strategy, which targeted indoor-resting individuals, as this species shows outdoor host-seeking and resting behaviors. In addition, the sites at Busia and Bungoma are in highlands, far from lakes, and in other studies a low number of collected *An. arabiensis* was attributed to the distance of the sampling site from lakes [14], temporal and spatial variation [16], and/or changes in ecological factors [24]. While sibling species of the genus *Anopheles* have been found to live in sympatry, *An. arabiensis* prefers areas with high temperatures and low relative humidity, such as lowlands [12, 16]. The observation that *An. gambiae* s.l. was the predominant malaria vector in these highland sites of Kenya followed by *An. funestus* s.l. corroborates previous findings. Although *Microsporidia MB* was primarily detected in *An. gambiae* s.s. in the present study, extensive sampling that targets *An. funestus* in the Kenyan highlands may allow for the detection of *Microsporidia MB* in these hosts, as this symbiont has been detected in this mosquito species in the lowlands of Ahero [11]. It is possible that scaled-up vector control strategies, such as indoor-based interventions [1, 3], may partly influence mosquito behaviors, including, but not limited to, their preferences for outdoor and/or indoors resting and biting [18]. Thus the use of a single approach for mosquito collection, such as indoor aspiration, may influence the measured species abundance and also the *Microsporidia MB* positivity rate.

Herren et al. [10] attributed a low *Microsporidia MB* infection rate in *An. arabiensis* (0–9%) to several factors. Firstly, the prevalence of *Microsporidia MB* was found to correlate strongly with season, with infection rates increasing immediately after the rains [10]. While we did not examine seasonal fluctuations, it is possible that changes in the infection rates in *An. gambiae* s.s. found here may also have been related to the these. Secondly, *An. gambiae* s.s. from western Kenya have been found to have insecticide-resistance traits and associations with other microbes that may have modulatory effects on their insecticide resistance [26, 27]. Thus, it is possible that the *An. gambiae* s.s. examined in the present study have multiple insecticide-resistance traits, and the circulation of insecticide-resistant phenotypes of this host may influence *Microsporidia MB* infection rates in the geographical locations of the present study. Third, changes in the infection rates of maternally inherited symbionts such as *Wolbachia* have been attributed to changes in temperature, which also affect the densities of specific strains of *Wolbachia* [28, 29]. Although the effects of temperature on the intensity of *Microsporidia MB* infection and transmission in mosquito hosts have not been studied*,* it is possible that chemical interactions similar to those observed in *Wolbachia* infections [28, 30], or even unique ones, could be associated with the maternal inheritance of symbionts such as *Microsporidia MB* in mosquitoes. Further investigation of these hypotheses could help to explain why the prevalence of *Microsporidia MB* in the field remains low despite the observed high transmission rate of *Microsporidia MB* from field-collected mothers to their offspring.

The results showed that the development of *Microsporidia MB* in the midgut and reproductive tissues does not impose fitness costs on *An. gambiae* s.s. *Anopheles gambiae* s.s. females infected with *Microsporidia MB* transmitted the infection to their offspring at a rate of approximately 69% (range 0–100%). Low *Microsporidia MB* intensities were observed in two of the females that did not transmit the infection to their offspring. A high infection intensity of *Microsporidia MB* was observed in the reproductive tissues of 5- to 7-day-old G_1_ adults relative to their gut tissues, although in females the intensity of infection in the gut and reproductive tissues did not significantly differ. These results suggest that the gut tissues may be an important reservoir for *Microsporidia MB* in *An. gambiae* s.s. A weak positive correlation between *Microsporidia MB* infection intensities in G_0_ females and their G_1_ offspring was observed. It is possible that infection of the reproductive tissues with *Microsporidia MB* may influence its transmission to offspring, albeit slightly [10], but it is unknown if infection intensities in the midgut are related to the proliferation of *Microsporidia MB* in reproductive tissues.

Infection of *An. gambiae* s.s. with *Microsporidia MB* did not have a negative effect on several of its life history traits. For instance, females infected with *Microsporidia MB* and non-infected females laid an equivalent number of eggs, and the larvae infected with *Microsporidia MB* had a marginally significant faster mean development time. Mortality did not differ significantly between *Microsporidia MB*-infected and non-infected larvae, and infected adults had a similar lifespan to non-infected ones. Whether *Microsporidia MB* infection results in changes in these parameters when mosquitoes are exposed to nutritional and temperature-related stress is unknown. Previous reports showed that *An. arabiensis* [10] and *An. gambiae* s.s. larvae infected with *Microsporidia MB* had a slightly faster pupal development time than non-infected larvae, but the overall survival rates did not differ between them. Although the larval survival rates of *An. gambiae* s.s. did not differ between the *Microsporidia MB*-infected and non-infected individuals in the present study, future studies should investigate if *Microsporidia MB* infection load influences pupation rate and subsequent survival.

The faster development rate of infected pupae suggests that *Microsporidia MB* may influence nutrient availability and host metabolic processes [10]. A distortion in the sex ratio favoring females has been previously observed in symbionts that utilize transovarial transmission, such as* Dictyocoela* microsporidia [31]. The lack of distortion in the sex ratio of *An. gambiae* s.s. adults infected with *Microsporidia MB* agrees with previous observations in *An*. *arabiensis* [10]. The sex ratio was determined for the emerging adults, but whether there was a similar sex ratio in the eggs laid by females with different infection loads is unknown.

Overall, our findings suggest that *Microsporidia MB* is avirulent in two species of the *An. gambiae* species complex, *An. arabiensis and An. gambiae* s.s., in contrast to other mosquito-associated microsporidians that have been found to be virulent in mosquitoes [32–37]. It was shown that *Microsporidia MB* is transmitted from infected females to their offspring and also horizontally between adults. This is significant, since *Microsporidia MB*-infected males derived from infected females could be used in a strategy for the dissemination of *Microsporidia MB*, which would thus avoid the need to release biting females.

## Conclusions

The finding that the characteristics of *Microsporidia MB* infection in *An. gambiae* s.s. are similar to those reported for *An. arabiensis* [9, 10] supports the prospect of developing a *Microsporidia MB*-based malaria transmission-blocking strategy for both of these important vector species. Future studies will need to specifically investigate whether *Microsporidia MB* protects *An. gambiae* s.s. against *P. falciparum*. In addition, the sequencing of *Microsporidia MB* from both of these species should reveal if there are diverse *Microsporidia* *MB* strains that infect different members of the *An. gambiae* species complex. It would also be of interest to determine if *Microsporidia MB* can be transmitted between members of the *An. gambiae* species complex.

### Supplementary Information

**Additional file 1: Figure S1.** Sex ratio of *Microsporidia MB-*infected *Anopheles gambiae *s.s. progeny reared in pools. **A** Bar plots representing the sex ratio of *Microsporidia MB*-infected offspring and non-infected counterparts reared in pools (*n* = 6, where a pool consists of approximately seven individual females). **B** Bar plots representing the mean prevalence of* Microsporidia MB* transmitted to* An. gambiae* s.s. offspring, reared in pools, by females. Error bars represent the SEM percentage (* *P* < 0.05).

**Additional file 2: Figure S2. ***Microsporidia MB* infection intensities in *An. gambiae* s.s. influence the rate of transmission to offspring. **A** Correlation of G_o_ *Microsporidia MB* intensities with average G_1_ *Microsporidia MB* densities [*r*^2^ = 0.1276, *P* = 0.268, number of broods = 18]. **B** Correlation between G_0_ *Microsporidia MB* intensities and *Microsporidia MB* transmission to offspring (*r*^2^ = 0.625, *P* = 0.0056, *n* = 18).

**Additional file 3: Table S1.** Annual prevalence of *Microsporidia MB* in *Anopheles gambiae *s.l. Values indicate yearly (2021–2022) abundance (%) of *Microsporidia MB* assessed from the DNA of individual female mosquitoes sampled from Bungoma and Busia.

**Additional file 4: Table S2.** Horizontal transmission of *Microsporidia MB* between *Anopheles gambiae* s.s. adults reared together in cages. Values indicate intensities of *Microsporidia MB *infection in donor male/female and recipient male/female* An. gambiae* s.s. individuals reared together in a cage.

## Data Availability

All of the datasets supporting the conclusions of this article are included within the article.

## Abbreviations

KEMRI: Kenya Medical Research Institute
PCR: Polymerase chain reaction
qPCR: Quantitative polymerase chain reaction
