# Supplementary figures and images for: Microsporidia MB in the primary malaria vector Anopheles gambiae sensu stricto is avirulent and undergoes maternal and horizontal transmission
Source: Parasit Vectors. 2023 Sep 25;16:335. doi: 10.1186/s13071-023-05933-8 (PMC10519057; doi:10.1186/s13071-023-05933-8)

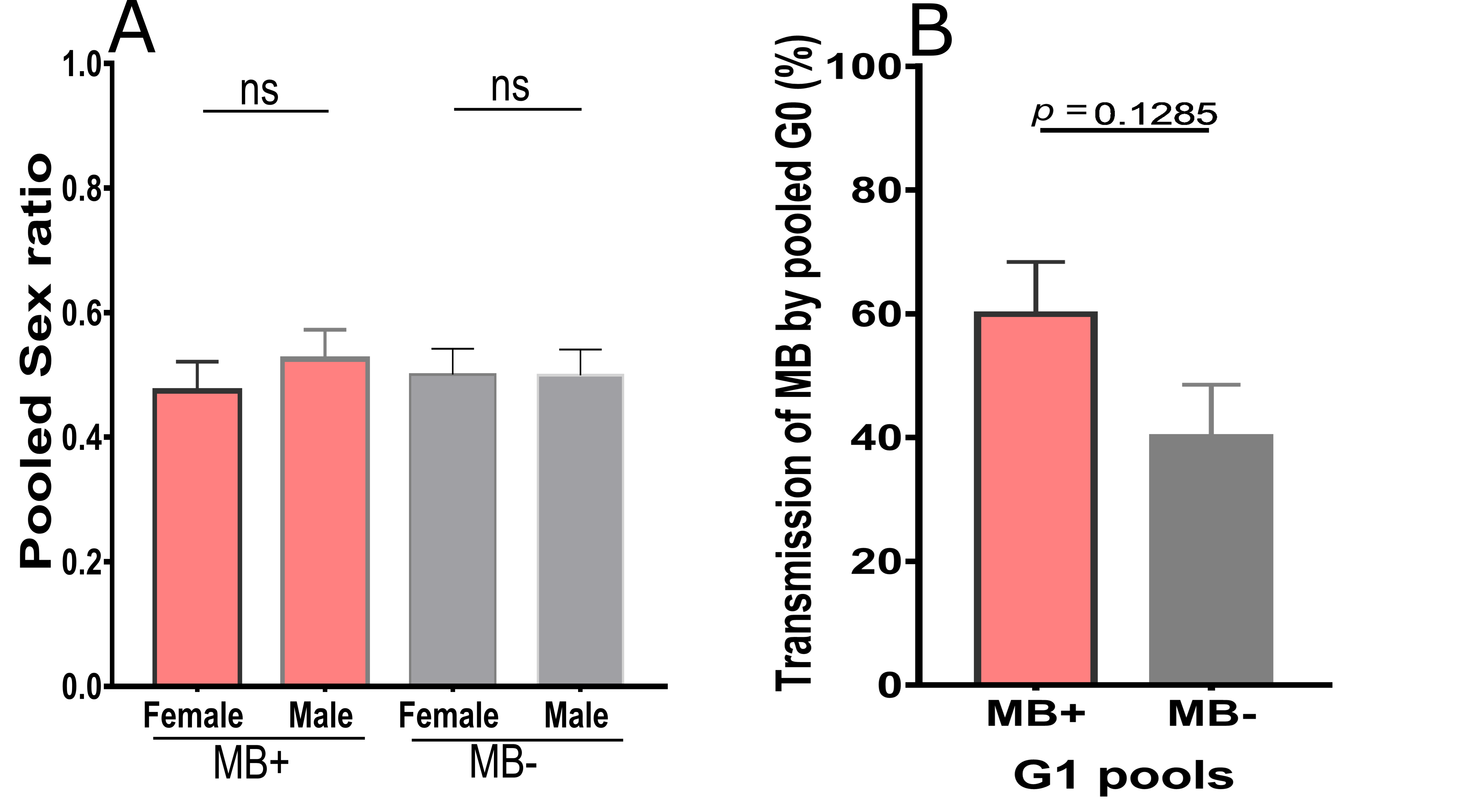

Supplement: Supplementary file 1 — Additional file 1: Figure S1. Sex ratio of Microsporidia MB-infected Anopheles gambiae s.s. progeny reared in pools. A Bar plots representing the sex ratio of Microsporidia MB-infected offspring and non-infected counterparts reared in pools (n = 6, where a pool consists of approximately seven individual females). B Bar plots representing the mean prevalence of Microsporidia MB transmitted to An. gambiae s.s. offspring, reared in pools, by females. Error bars represent the SEM percentage (* P < 0.05). [file 13071_2023_5933_MOESM1_ESM.tiff]

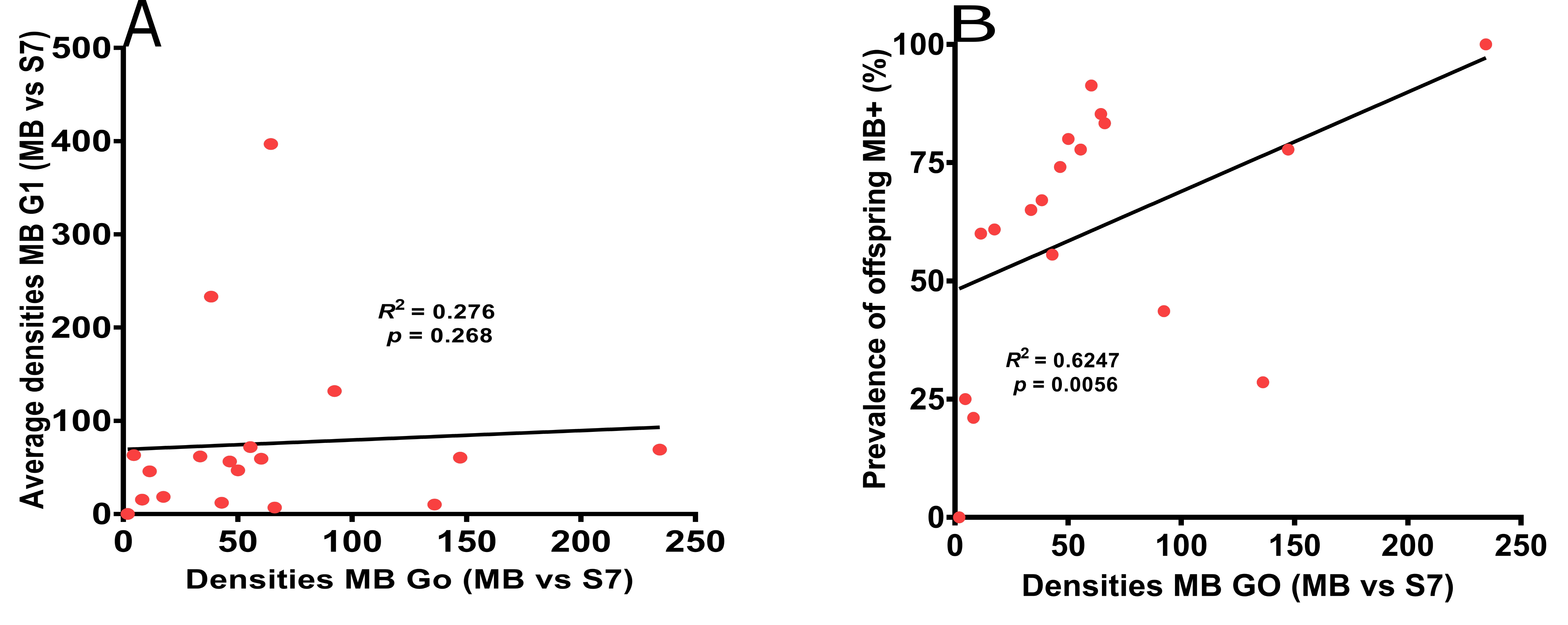

Supplement: Supplementary file 2 — Additional file 2: Figure S2. Microsporidia MB infection intensities in An. gambiae s.s. influence the rate of transmission to offspring. A Correlation of Go Microsporidia MB intensities with average G1 Microsporidia MB densities [r2 = 0.1276, P = 0.268, number of broods = 18]. B Correlation between G0 Microsporidia MB intensities and Microsporidia MB transmission to offspring (r2 = 0.625, P = 0.0056, n = 18). [file 13071_2023_5933_MOESM2_ESM.tiff]
