# Supplementary material for: Microsporidia MB in the primary malaria vector Anopheles gambiae sensu stricto is avirulent and undergoes maternal and horizontal transmission
Source: Parasit Vectors. 2023 Sep 25;16:335. doi: 10.1186/s13071-023-05933-8 (PMC10519057; doi:10.1186/s13071-023-05933-8)
Supplement: Supplementary file 3 — Additional file 3: Table S1. Annual prevalence of Microsporidia MB in Anopheles gambiae s.l. Values indicate yearly (2021–2022) abundance (%) of Microsporidia MB assessed from the DNA of individual female mosquitoes sampled from Bungoma and Busia. [file 13071_2023_5933_MOESM3_ESM.docx]

Additional file: Table S1: Evidence of *Microsporidia* MB in *Anopheles gambiae* s.s.

| **Year of collection** | **Development stage collected** | **Sex** | **DNA Sample (pooled or individuals** | **Study site** | ***An. gambiae* s.s.** | | | ***An. funestus* s.s.** | | | ***An. arabiensis*** | | |
| --- | --- | --- | --- | --- | --- | --- | --- | --- | --- | --- | --- | --- | --- |
|  |  |  |  |  | **Total number Screened** | **MB Positive number** | **Abundance (%)** | **Total number Screened** | **MB Positive number** | **Abundance (%)** | **Total number Screened** | **MB Positive number** | **Abundance (%)** |
| 2020 | Adults | Females | Individuals | Busia | 1448 | 18 | 1.710 | 285 | 0 |  | 52 | 0 | 0.901 |
| 2021 | Adults | Females | Individuals | Busia | 649 | 6 |  | 303 | 0 | 0 | - | - |  |
| 2022 | Adults | Females | Individuals | Busia | 2464 | 54 |  | 181 | 0 |  | 281 | 3 |  |
| 2020 | Adults | Females | Individuals | Bungoma | 370 | 1 | 0.198 | 285 | 0 | 0 | - | - | 0 |
| 2021 | Adults | Females | Individuals | Bungoma | 136 | 0 |  | 380 | 0 |  | - | - |  |
|  |  |  |  | Total | 5067 | 79 |  | 1434 | 0 |  | 333 | 3 |  |
|  |  |  |  | MB Positivity rate /site |  |  | 1.559 |  |  | 0 |  |  | 0.901 |
|  |  |  |  |  |  |  | ~1.56 |  |  | ~0.00 |  |  | ~0.91 |
