# Supplementary material for: Microsporidia MB in the primary malaria vector Anopheles gambiae sensu stricto is avirulent and undergoes maternal and horizontal transmission
Source: Parasit Vectors. 2023 Sep 25;16:335. doi: 10.1186/s13071-023-05933-8 (PMC10519057; doi:10.1186/s13071-023-05933-8)
Supplement: Supplementary file 4 — Additional file 4: Table S2. Horizontal transmission of Microsporidia MB between Anopheles gambiae s.s. adults reared together in cages. Values indicate intensities of Microsporidia MB infection in donor male/female and recipient male/female An. gambiae s.s. individuals reared together in a cage. [file 13071_2023_5933_MOESM4_ESM.docx]

**Additional file: Table S2: Horizontal transmission of Microsporidia MB between An. gambiae s.s. adults reared together in cages.**

| **Experiment** | **# of donor mates in cage** | **# of confirmed MB+ donor mates in the cage** | **Total exposed screened** | **Donor 1 intensity** | **Donor 2 intensity** | **Donor 3 intensity** | **Donor 4 intensity** | **Donor 5 intensity** | **Donor 6 intensity** | **Donor 7 intensity** |
| --- | --- | --- | --- | --- | --- | --- | --- | --- | --- | --- |
| Male to Female | |  |  |  |  |  |  |  |  |  |
| BUR_KSMS 1 | 6 | 4 | 24 | 8.0032946 | 4.808316 | 63.294066 | 8.3362295 | NA | NA | NA |
| BUR_KSMS 2 | 8 | 7 | 34 | 1.2258022 | 3.538059 | 5.7570451 | 10.965804 | 0.3481739 | 6.1658063 | 10.02655 |
| BUR_KSMS 3 | 5 | 1 | 15 | 10.575466 | NA | NA | NA | NA | NA | NA |
| BUR_KSMS 4 | 21 | 19 | 44 | 26.540992 | 19.96888 | 245.49992 | 110.91252 | 197.79728 | 36.926989 | 5.530842 |
| BUR_KSMS 5 | 11 | 9 | 29 | 1.4042662 | 4.229069 | 20.300458 | 52.764212 | 409.68845 | 355.86827 | 118.8288 |
| BUR_KSMS 11 | 3 | 2 | 26 | 12.625816 | 7.729092 | NA | NA | NA | NA | NA |
| BUR_KSMS 12 | 2 | 1 | 21 | 2.8132617 | NA | NA | NA | NA | NA | NA |
| BUR_KSMS 14 | 2 | 2 | 12 | 1.1620017 | 0.710227 | NA | NA | NA | NA | NA |
| BUR_KSMS 15 | 2 | 2 | 27 | 38.027816 | 0.11871 | NA | NA | NA | NA | NA |
| BUR_KSMS 22 | 4 | 1 | 31 | 4.5719256 | NA | NA | NA | NA | NA | NA |
| BUR_KSMS 23 | 6 | 1 | 36 | 1.0526719 | NA | NA | NA | NA | NA | NA |
| Female to Male | |  |  |  |  |  |  |  |  |  |
| BUR_KSMS 6 | 4 | 4 | 16 | 0.1881144 | 254.7827 | 105.85124 | 5.9498411 | NA | NA | NA |
| BUR_KSMS 7 | 4 | 3 | 19 | 11.233807 | 11.78256 | 1.1102048 | NA | NA | NA | NA |
| BUR_KSMS 8 | 14 | 3 | 37 | 13.747496 | 8.8E-06 | NA | NA | NA | NA | NA |
| BUR_KSMS 9 | 7 | 2 | 24 | 4.5978201 | 3.076828 | NA | NA | NA | NA | NA |
| BUR_KSMS 10 | 7 | 5 | 24 | 2.5498819 | 6.125434 | 3.5096678 | 10.076895 | NA | NA | NA |
| BUR_KSMS 16 | 3 | 1 | 26 | 2.6325163 | NA | NA | NA | NA | NA | NA |
| BUR_KSMS 17 | 5 | 1 | 17 | 7.5206272 | NA | NA | NA | NA | NA | NA |
| BUR_KSMS 18 | 2 | 1 | 22 | 21.829099 | NA | NA | NA | NA | NA | NA |
| BUR_KSMS 19 | 2 | 2 | 31 | 43.387927 | 24.98367 | NA | NA | NA | NA | NA |

**Additional file: Table S2 continues**

| **Donor 8 intensity** | **Donor 9 intensity** | **remaining Donors average intensity** | **# recipients acquiring MB infection** | **# recipients didn’t acquire MB infection** | **% Transmission** | **Recipient 1 intensity** | **Recipient 2 intensity** | **Recipient 3 intensity** |
| --- | --- | --- | --- | --- | --- | --- | --- | --- |
|  |  |  |  |  |  |  |  |  |
| NA | NA | NA | 3 | 21 | 12.5 | 0.047655 | 0.1502304 | 67.756806 |
| NA | NA | NA | 3 | 31 | 8.823529412 | 18.957415 | 131.44081 | 23.859959 |
| NA | NA | NA | 0 | 15 | 0 | NA | NA | NA |
| 28.018561 | 17.829624 | 40.03082657 | 3 | 44 | 6.382978723 | 1.4173024 | 53.280165 | 7.4193393 |
| 15.462887 | 1.2500258 | NA | 1 | 28 | 3.448275862 | 0.5542516 | NA | NA |
| NA | NA | NA | 0 | 26 | 0 | NA | NA | NA |
| NA | NA | NA | 0 | 21 | 0 | NA | NA | NA |
| NA | NA | NA | 0 | 12 | 0 | NA | NA | NA |
| NA | NA | NA | 0 | 27 | 0 | NA | NA | NA |
| NA | NA | NA | 0 | 31 | 0 | NA | NA | NA |
| NA | NA | NA | 0 | 36 | 0 | NA | NA | NA |
|  |  |  |  |  |  |  |  |  |
| NA | NA | NA | 1 | 15 | 6.25 | 10.360733 | NA | NA |
| NA | NA | NA | 0 | 19 | 0 | NA | NA | NA |
| NA | NA | NA | 0 | 37 | 0 | NA | NA | NA |
| NA | NA | NA | 0 | 24 | 0 | NA | NA | NA |
| NA | NA | NA | 0 | 24 | 0 | NA | NA | NA |
| NA | NA | NA | 0 | 12 | 0 | NA | NA | NA |
| NA | NA | NA | 0 | 17 | 0 | NA | NA | NA |
| NA | NA | NA | 0 | 21 | 0 | NA | NA | NA |
| NA | NA | NA | 0 | 24 | 0 | NA | NA | NA |
